# Supplementary material for: Hepatic Epithelioid Hemangioendothelioma in a Dog
Source: Animals (Basel). 2024 Apr 25;14(9):1302. doi: 10.3390/ani14091302 (PMC11083527; doi:10.3390/ani14091302)
Supplement: Supplementary file 1 [file animals-14-01302-s001.zip › Supplementary Figure S1 legend.pdf]

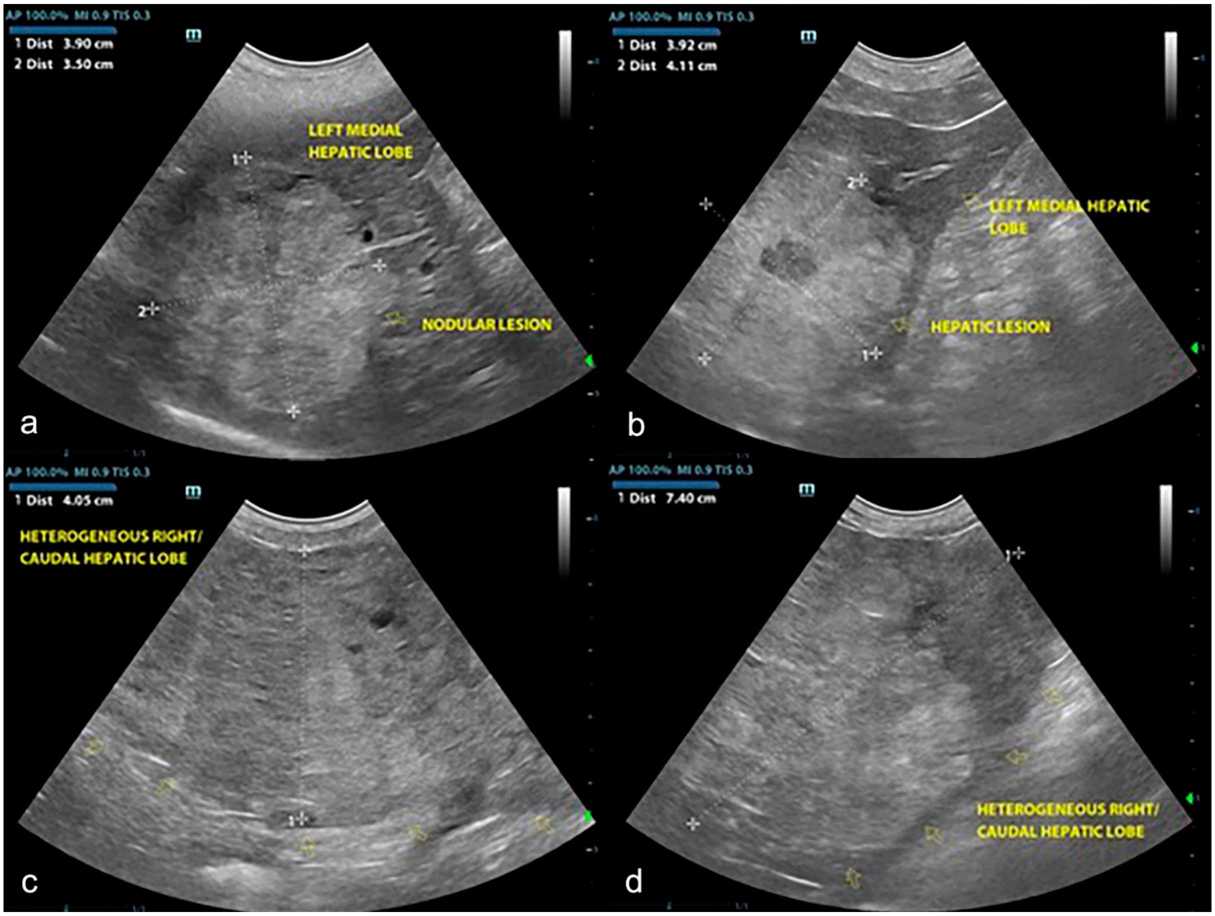

Figure S1. Ultrasound of epithelioid hemangioendothelioma, liver, dog. **a)** Left lobe with expansive nodular lesion, **b)** deforming the profile, hyperechoic at defined margins. **c)** Right hepatic lobes of increased size with rounded profile, dysmogeneous parenchyma due to the presence of hyperechoic not defined margin areas. **d)** Remaining parenchyma with coarse structure.
