# Supplementary material for: Hepatic Epithelioid Hemangioendothelioma in a Dog
Source: Animals (Basel). 2024 Apr 25;14(9):1302. doi: 10.3390/ani14091302 (PMC11083527; doi:10.3390/ani14091302)
Supplement: Supplementary file 1 [file animals-14-01302-s001.zip › Supplementary Table S1.pdf]

Table S1. Biochemical parameters in dog with hepatic epithelioid hemangioendothelioma.

Parameter	Value	Unit	Reference range
GLU	106	mg/dL	74-120
ALP	47	U/L	16-119
GGT	0	U/L	0-11
T.BIL	0.33	mg/dL	0-0.45
AST	31	U/L	15-44
ALT	49	U/L	22-78
ALB	3.7	g/DL	2.8-4
